# EBV-associated recurrent Hodgkin's disease after renal transplantation

**DOI:** 10.1111/j.1432-2277.2006.00273.x

**Published:** 2006-04

**Authors:** Katherine H Flanagan, Daniel C Brennan

**Affiliations:** Washington University School of Medicine, Department of Internal Medicine, Renal Division St Louis, MO, USA

**Keywords:** Epstein–Barr virus, Hodgkin's disease, kidney transplant, post-transplant lymphoproliferative disease, recurrence

## Abstract

Hodgkin's disease is recognized as part of the spectrum of post-transplantation lymphoproliferative disorders (PTLD), although it is still an uncommon *de novo* malignancy in this population. Epstein–Barr virus (EBV) has been linked to both post-transplant non-Hodgkin's lymphomas and Hodgkin's disease. We report a case of recurrent Hodgkin's disease in a patient who received a renal transplant in childhood and later developed EBV-associated Hodgkin's disease with remission after chemotherapy until subsequent relapse 9 years later that was successfully treated. To our knowledge, this is the first report of recurrent Hodgkin's disease in a transplant recipient. We briefly discuss the pathogenesis of and risk factors for EBV-related PTLD, utility of EBV load surveillance, and the options for treatment of PTLD including immunosuppression reduction, antiviral therapy, anti-CD20 monoclonal antibodies, cytotoxic T cells, and the possible roles of interferon-α and rapamycin.

## Case report

The patient is a 27-year-old white man who received a living-related donor transplant from his father at 11 years of age in 1986 for end stage renal disease secondary to posterior urethral valves. The patient was seronegative for cytomegalovirus (CMV) and Epstein–Barr virus (EBV) prior to transplantation. His father's EBV and CMV status were unavailable. The transplant course was complicated by immediate postoperative acute renal artery thrombosis, which required transplant nephrectomy. He then received a deceased donor renal transplant 10 days later. Maintenance immunosuppression consisted of cyclosporine (Sandimmune formulation), azathioprine, and prednisone. He received 12 doses of Minnesota equine antilymphocyte immunoglobulin (MALG) for induction followed by 10 doses of murine anti-CD3 monoclonal antibody (OKT3) for two episodes of acute graft rejection over the 7 weeks following the transplant. The deceased donor's EBV and CMV status were unavailable from medical records. The patient developed acute infectious mononucleosis 2 months after transplantation demonstrated by positive EBV serology. He was treated with acyclovir and the infectious mononucleosis resolved.

At the age of 18, he presented with 5 months of fever, weight-loss and night sweats, and was found to have axillary adenopathy, splenomegaly and bulky right mediastinal adenopathy. A right anterior, thoracotomy and lymph node biopsy demonstrated Hodgkin's disease, nodular sclerosing subtype. Hodgkin's–Reed–Sternberg (HRS) cells were strongly positive for CD30 and EBV-latent membrane protein (LMP)-1. He was staged as Hodgkin's disease-IIIB. His EBV serology demonstrated elevated antiviral capsid antigen (VCA) IgG titers and positive anti-VCA IgM, consistent with reactivation of EBV infection.

He was given six cycles of adriamycin, bleomycin, vinblastine, and dacarbazine (ABVD) chemotherapy, without significant side effects, and his Hodgkin's Disease went into remission. His serum creatinine after therapy was 2.3 mg/dl. Azathioprine was discontinued for leukopenia, and cyclosporine and prednisone continued until he developed renal insufficiency, with a serum creatinine of 7.8 mg/dl 5-years later. A biopsy showed severe chronic rejection. The patient was changed from Sandimmune to Neoral, and mycophenolate mofetil (MMF) was added. Notably, his CMV IgG and IgM were positive at this time, indicating seroconversion in the preceding 2 years since his CMV status had last been determined. His renal function improved to his baseline serum creatinine of 2.4 mg/dl.

Four years later, serum creatinine was 3.2 mg/dl and a biopsy demonstrated chronic allograft nephropathy with moderate interstitial fibrosis. Neoral was changed to tacrolimus, and he continued on MMF and prednisone. His creatinine stabilized at a new baseline of 2.7 mg/dl.

Nine years after remission, the patient was found to have hypercalcemia of 15.4 mg/dl associated only with irritability. He was found to have retroaortic and retrocrural lymphadenopathy with mild paratracheal lymphadenopathy on Computed Tomography scan. Excisional biopsy showed Hodgkin's disease-IIIA of mixed cellularity subtype. The HRS cells were strongly positive for CD15, CD30, and EBV LMP-1. The patient's serology showed elevated anti-VCA IgM and anti-VCA IgG titers.

Tacrolimus and MMF were discontinued, and ABVD and valganciclovir commenced. He achieved remission of his recurrent HD with chemotherapy and immunosuppressive withdrawal. Adriamycin was subsequently changed to doxorubicin to prevent cardiotoxicity, and bleomycin was discontinued from his regimen secondary to lung toxicity. His chronic renal insufficiency worsened, and he returned to hemodialysis 9 months after diagnosis of recurrent Hodgkin's Disease. At the time of his return to dialysis, he was found to have a positive hepatitis C antibody.

## Discussion

Although several case reports of Hodgkin's disease and Hodgkin-like PTLD (HL-PLTD) after organ transplantation have been published, no case of recurrent classic Hodgkin's disease has yet been reported [1]. Our patient is remarkable for late recurrent Hodgkin's disease after renal transplantation in association with reactivation of EBV infection, similar to his initial episode.

Epstein–Barr virus genomes have been detected in 60% of Hodgkin's disease in immunocompetent individuals, and EBV viremia is common with infectious mononucleosis [2]. Expression of EBV-encoded proteins in HRS is limited to the latency II pattern with EBNA1, LMP-1, 2A, 2B proteins, and EBV early RNAs (EBERS) [3]. The most important of the EBV genome products is an LMP-1 protein, which is highly expressed in HRS cells in EBV-associated Hodgkin's disease.

Latent membrane protein (LMP)-1 is an integral membrane protein that mimics a constitutively active CD40 receptor on B cells [4]. The LMP-1 is a target epitope for cytotoxic T cell recognition for EBV-infected cells, and transplant patients are unable to recognize and destroy these cells because of immunosuppression. Certain immunosuppressants increase the likelihood of PTLD by the suppression of cytotoxic T cell surveillance.

Other risk factors for the development of PTLD include intensity of immunosuppression, EBV seronegative status, young age, induction therapy with antilymphocyte agents such as OKT3, and infection with CMV or hepatitis C [5, 6]. Our patient received OKT3 prior to his first episode of Hodgkin's disease, had been treated with tacrolimus for several years before recurrence of Hodgkin's disease, and had become CMV and hepatitis C seropositive in the interval between his first and second episodes of Hodgkin's disease.

Monitoring EBV loads may allow for detection of early PTLD, but the utility of following EBV viral loads is unclear. Assays are not standardized, and it is not known which tissue to sample, or when to test [7]. In pediatric organ transplant recipients, high viral loads are sensitive but not specific for the development of PTLD. In adults, high EBV levels are not sensitive but are specific for the development of PTLD. EBV-associated PTLD has been demonstrated in patients with low or undetectable EBV loads, and elevated EBV viral loads develop in patients with EBV-associated viral syndromes [8]. Monitoring viral loads during the treatment of EBV may aid management. Persistently elevated titers necessitate alternative treatment.

A novel method for detection of EBV-mediated transformation of B-cells was recently described in which a spontaneous EBV B-cell transformation assay (SET) was used to monitor an EBV-specific immunity *in vivo*. A positive SET correlated with a decrease in EBV-peptide-specific CD8+ T cells and a higher rate of EBV replication and B-cell transformation in the absence of cytotoxic T-cell suppression. Reduction of immunosuppression resulted in negative SET assays, and this approach may allow for surveillance of EBV-mediated lymphoproliferation in transplant patients [9].

Treatment of PTLD includes reduction of immunosuppression, antiviral therapy, anti-CD20 monoclonal antibodies, intravenous immunoglobulin (IVIG), cytotoxic T cells, radiation and surgery. Reduction of immunosuppression is first-line therapy and enables native immune function to clear the EBV-viremia and associated PTLD [10].

The utility of antivirals for treatment is unclear. Most EBV-infected cells within PTLD lesions are in the latent phase of replication, and antivirals such as acyclovir and ganciclovir target the lytic phase. There is an increasing evidence, however, that the lytic viral phase plays a larger role in PTLD than previously thought [7].

The use of anti-B cell monoclonal antibodies is increasing. In a recent retrospective study of 30 solid organ transplant patients with PTLD at the Mayo Clinic, 15 CD20 and EBER-positive patients who did not respond to reduction of immunosuppression were treated with rituximab [11]. Rituximab was associated with a significantly improved survival by multivariate analysis. Complications included profound hypogammaglobulinemia, CMV reactivation with intestinal perforation, hepatitis B and C reactivation, and parvovirus-induced red blood cell aplasia [7].

Therapy with cytotoxic T cells for prevention and treatment of EBV-related PTLD has been investigated in solid organ transplant and bone marrow transplant (BMT) patients. Comoli *et al*. [12] developed autologous EBV-specific CTLs for 23 patients at high risk for PTLD because of elevated EBV DNA loads. Seven patients received EBV-specific CTLs and a stable decrease in EBV load was detected in five without graft function compromise, and in a 30-month follow-up no patients developed PTLD, even in the two patients who did not respond.

Interferon-α and rapamycin have also been used in treatment of PTLD. The potential for eliciting acute allograft rejection with interferon-α limits its use [1]. Rapamycin, a transplant immunosuppressant, has antiproliferative activity against EBV-associated PTLD *in vitro*, and inhibits the growth of solid tumors from EBV-infected B cell lines in a xenogeneic mouse model of PTLD [13]. Rapamycin may therefore be useful for both immunosuppression and treatment of EBV-associated PTLD but more data are needed.

Prophylaxis for PTLD with antivirals has been used. Despite a greater degree of immunosuppression in the ganciclovir group, prophylaxis with ganciclovir was more effective than acyclovir in a high-risk group of 198 patients, with solid organ transplants, who received OKT3 induction therapy. PTLD developed in 0.5% in the ganciclovir group and 3.9% in the acyclovir group [14]. Prophylaxis with ganciclovir may have been more effective, indirectly, as it is more effective than acyclovir against CMV.

In summary, we report the first case of recurrent Hodgkin's disease in a renal transplant patient with numerous risk factors for PTLD. Our case is notable for several features: (i) initial EBV seronegativity, (ii) subsequent conversion after an acute infectious mononucleosis infection early after transplantation, (iii) the subsequent development of Hodgkin's disease with successful treatment, (iv) and then recurrent Hodgkin's disease successfully treated. Both instances of Hodgkin's disease were associated with reactivation of EBV. Further investigation of the complex mechanisms promoting the EBV transformation of B cells in immunocompromised hosts is necessary, and options for treatment of PTLD continue to broaden.
